# A Higher-Than-Standard-Intensity International Normalized Ratio Goal for Patients Undergoing Mechanical Aortic Valve Replacement With Additional Thrombotic Risk Factors: Protocol for a Systematic Review and Meta-Analysis

**DOI:** 10.2196/73389

**Published:** 2025-07-10

**Authors:** Myung-Rho Kim, Taha Shaikh, Shawn Wang, Spencer Taylor, Vidhani Goel, Banveet Kaur Khetarpal, Chowdhury Ahsan, Kavita Batra

**Affiliations:** 1 Department of Internal Medicine Kirk Kerkorian School of Medicine University of Nevada, Las Vegas Las Vegas United States; 2 Kirk Kerkorian School of Medicine University of Nevada, Las Vegas Las Vegas, NV United States; 3 School of Public Health University of Nevada, Las Vegas Las Vegas, NV United States; 4 Division of Cardiovascular Medicine Kirk Kerkorian School of Medicine University of Nevada, Las Vegas Las Vegas, NV United States; 5 Office of Research and Department of Medical Education Kirk Kerkorian School of Medicine University of Nevada, Las Vegas Las Vegas, NV United States

**Keywords:** mechanical aortic valve replacement, MAVR, thromboembolic risk factors, thromboembolism, anticoagulation, international normalized ratio, warfarin, Coumadin, vitamin K antagonist

## Abstract

**Background:**

Lifelong anticoagulation therapy with vitamin K antagonists is recommended following mechanical aortic valve replacement (MAVR) to prevent valve thrombosis. Current guidelines recommend a standard international normalized ratio (INR) of 2.5 for patients with MAVR without additional thromboembolic risk factors, and a higher INR goal of 3.0 for those with conditions such as atrial fibrillation, prior thromboembolism, or left ventricular dysfunction. However, limited clinical evidence exists to guide anticoagulation intensity in this high-risk subgroup, necessitating a systematic review.

**Objective:**

We aimed to assess the safety and efficacy of higher-intensity INR goals (>3.0) compared to standard-intensity goals (approximately 2.5) in patients with MAVR with additional thromboembolic risk factors.

**Methods:**

This protocol describes a systematic review and meta-analysis following PRISMA (Preferred Reporting Items for Systematic Reviews and Meta-Analyses) 2020 guidelines. A comprehensive literature search will be conducted across PubMed, Embase, and the Cochrane Library for studies published before December 18, 2024. Eligible studies include randomized controlled trials (RCTs), cohort studies, and follow-up studies involving adult patients with MAVR on warfarin therapy, stratified by the presence of additional thromboembolic risk factors. Non–English-language studies, case reports, editorials, and animal studies will be excluded.

**Results:**

The review will synthesize existing data to compare the risks and benefits of intensified anticoagulation in patients with MAVR with additional thromboembolic risk factors. Data analysis and manuscript preparation are scheduled for July-August 2025.

**Conclusions:**

This study will provide critical evidence on INR management in high-risk patients with MAVR, potentially informing future updates to clinical guidelines and optimizing the balance between thromboembolic prevention and bleeding risk.

**International Registered Report Identifier (IRRID):**

PRR1-10.2196/73389

## Introduction

Severe aortic valvular disease, if left untreated, carries a mortality rate exceeding 90% and often necessitates surgical intervention through mechanical aortic valve replacement (MAVR) [1-3]. After surgery, patients require lifelong anticoagulation therapy with vitamin K antagonists, such as warfarin, to prevent prosthetic valve thrombosis.

Current guidelines differ on the target international normalized ratio (INR) for patients with MAVR, especially those with additional thromboembolic risk factors. While the 2020 American College of Cardiology/American Heart Association (ACC/AHA) guidelines recommend an INR goal of 3.0 for high-risk patients, the 2012 American College of Chest Physicians (ACCP) guidelines continue to advise a standard INR of 2.5 [4]. These inconsistencies highlight the ongoing debate regarding optimal anticoagulation intensity in this subgroup.

The majority of MAVR candidates already exhibit structural cardiac changes, such as left ventricular hypertrophy, chamber dilation, or atrial fibrillation due to longstanding pressure overload [5,6]. These features inherently increase the risk of thromboembolism, suggesting that many MAVR recipients may require more intensive anticoagulation [7]. However, balancing thrombotic protection with bleeding risk is critical, and existing evidence is insufficient to guide personalized INR targets.

To date, only one multicenter retrospective study has evaluated anticoagulation strategies in patients with MAVR with thromboembolic risk factors, reporting fewer bleeding and thromboembolic events with standard-intensity therapy compared to higher-intensity regimens [8]. Despite its important insights, this single study is not sufficient to inform clinical guidelines.

Notably, there is currently no systematic review or meta-analysis specifically focused on patients with MAVR with additional thromboembolic risk factors. While prior studies have explored INR goals in broader MAVR populations, they have often failed to stratify outcomes by thromboembolic risk status [9-13]. Therefore, a targeted synthesis of existing evidence is needed to evaluate whether higher INR goals provide additional benefit or pose increased harm for this population.

Our systematic review aims to fill this gap by examining the safety and efficacy of intensified anticoagulation in patients with MAVR with elevated thromboembolic risk. These findings have the potential to inform future guideline updates and guide individualized management strategies for this high-risk group.

## Methods

### Ethical Considerations and Protocol Registration

Institutional review board approval was not required for this systematic review, as it did not involve patient interaction and was conducted using publicly available, published data. To maintain project integrity, it was registered with PROSPERO (registration CRD42025639037, registered on January 27, 2025). PROSPERO is an international database where systematic reviews across various fields, including health care, can be registered to prevent duplication and minimize reporting bias by comparing the protocol with the final review.

### Review Questions

Guided by the population, exposure, comparison, outcome, and study design (PECOS) framework [14], this study was developed to answer the following question: Does a higher than standard-intensity INR goal benefit patients with MAVR and additional thrombotic risk factors?

### Inclusion and Exclusion Criteria

The systematic review will include case studies, randomized controlled trials (RCTs), and single-arm studies, while the meta-analysis will primarily consist of RCTs and follow-up studies. Only English-language articles published before December 18, 2024, will be included. Single-patient case reports, abstract-only articles, animal studies, commentaries, position papers, opinions, and editorials will be excluded, as will other meta-analyses and systematic reviews.

The study populations will comprise patients older than 18 years with a prior MAVR who are receiving warfarin therapy. A specific INR goal was not defined in order to expand the inclusion criteria, thereby maximizing the sample size and statistical power. Patients younger than 18 years or not receiving warfarin therapy will be excluded. Additionally, patients with bioprosthetic aortic valve replacement (AVR) will be excluded, as they do not require warfarin therapy. Non-bileaflet mechanical valves will also excluded due to the higher thromboembolic risks associated with single-leaflet AVRs compared to bileaflet prostheses [15,16]. Only studies with a patient population meeting the above criteria will be included in the systematic review. Among these, we will include patients with and without additional thromboembolic risk factors. Specifically, the control group will include adult patients (aged ≥18 years) with MAVR who receive warfarin therapy and do not exhibit any of the following predefined thromboembolic risk factors: atrial fibrillation, prior venous thromboembolism, hypercoagulable states (eg, antiphospholipid syndrome or inherited thrombophilias), severe left ventricular systolic dysfunction (ejection fraction <40%), left atrial dimension >50 mm, spontaneous echocardiographic contrast in the left atrium, significant vascular disease, or current estrogen replacement therapy. Older-generation prostheses or non-bileaflet devices will be excluded due to practical concerns. This definition ensures a consistent comparator population across studies and enhances the reproducibility and interpretability of the systematic review and meta-analysis. Textbox 1 summarizes the inclusion and exclusion criteria.

**Inclusion and exclusion criteria guided by the population, exposure, comparison, outcome, and study design (PECOS) framework.**

**Inclusion criteria**

Population: age ≥18 years; mechanical aortic valve replacement (MAVR); presence of ≥1 thromboembolic risk factor (eg, atrial fibrillation, venous thromboembolism, hypercoagulability, ejection fraction <40%)

Exposure: warfarin-based anticoagulation with international normalized ratio goal defined or reported

Comparison: patients with MAVR receiving warfarin without thromboembolic risk factors: no atrial fibrillation, no venous thromboembolism, no hypercoagulability, ejection fraction ≥40%, left atrial dimension ≤50 mm, no spontaneous echo contrast, no significant vascular disease, not receiving estrogen therapy

Outcomes: primary outcomes include major or minor thromboembolic and bleeding events; secondary outcomes include all-cause mortality, prosthetic endocarditis, valve reoperation, and hemolytic anemia

Study design: randomized controlled trials, observational studies, single-arm studies in English

**Exclusion criteria**

Population: age <18 years; not receiving warfarin therapy; bioprosthetic aortic valve replacement; non-bileaflet mechanical valve

Outcomes: studies lacking extractable clinical outcome data

Study design: case reports, editorials, commentaries, conference abstracts, prior systematic reviews or meta-analyses, non–English-language articles

### Informational Sources and Search Strategy

Bibliographic databases, including PubMed, Cochrane, and Embase, were searched from the initial development through December 18, 2024. The search strategy was developed by an expert medical librarian and adheres to the guidelines outlined by the Peer-Reviewed Electronic Search Strategy (PRESS). Initially, the search strategy was designed for the PubMed database using search criteria specific to that platform. It was then adapted for use with other databases. The detailed search strategy is provided in Table 1.

**Table 1:** The search strategy. All databases were searched from inception to December 18, 2024.

Database and query number			Query		Results, n
**PubMed**					
	#1	INR		20,246	
	#2	International Normalized Ratio		13,833	
	#3	Warfarin		35,162	
	#4	Coumadin		35,662	
	#5	Vitamin k antagonist		11,315	
	#6	#1 OR #2 OR #3 OR #4 OR #5		56,899	
	#7	Mechanical aortic valve replacement		4375	
	#8	MAVR		55	
	#9	#7 OR #8		4405	
	#10	#6 AND #9		341	
**Embase**					
	#1	INR		61,976	
	#2	International Normalized Ratio		55,005	
	#3	Warfarin		117,951	
	#4	Coumadin		5260	
	#5	Vitamin k antagonist		9682	
	#6	#1 OR #2 OR #3 OR #4 OR #5		167,310	
	#7	Mechanical aortic valve replacement		7589	
	#8	MAVR		82	
	#9	#7 OR #8		7626	
	#10	#6 AND #9		1114	
**Cochrane Library^a^**					
	#1	INR		4847	
	#2	International Normalized Ratio		3434	
	#3	Warfarin		5802	
	#4	Coumadin		234	
	#5	Vitamin k antagonist		909	
	#6	#1 OR #2 OR #3 OR #4 OR #5		10,974	
	#7	Mechanical aortic valve replacement		261	
	#8	MAVR		3	
	#9	#7 OR #8		262	
	#10	#6 AND #9		61	

^a^The search for the Cochrane Library was limited to “all text.”

### Screening and Data Management

**Figure 1:**
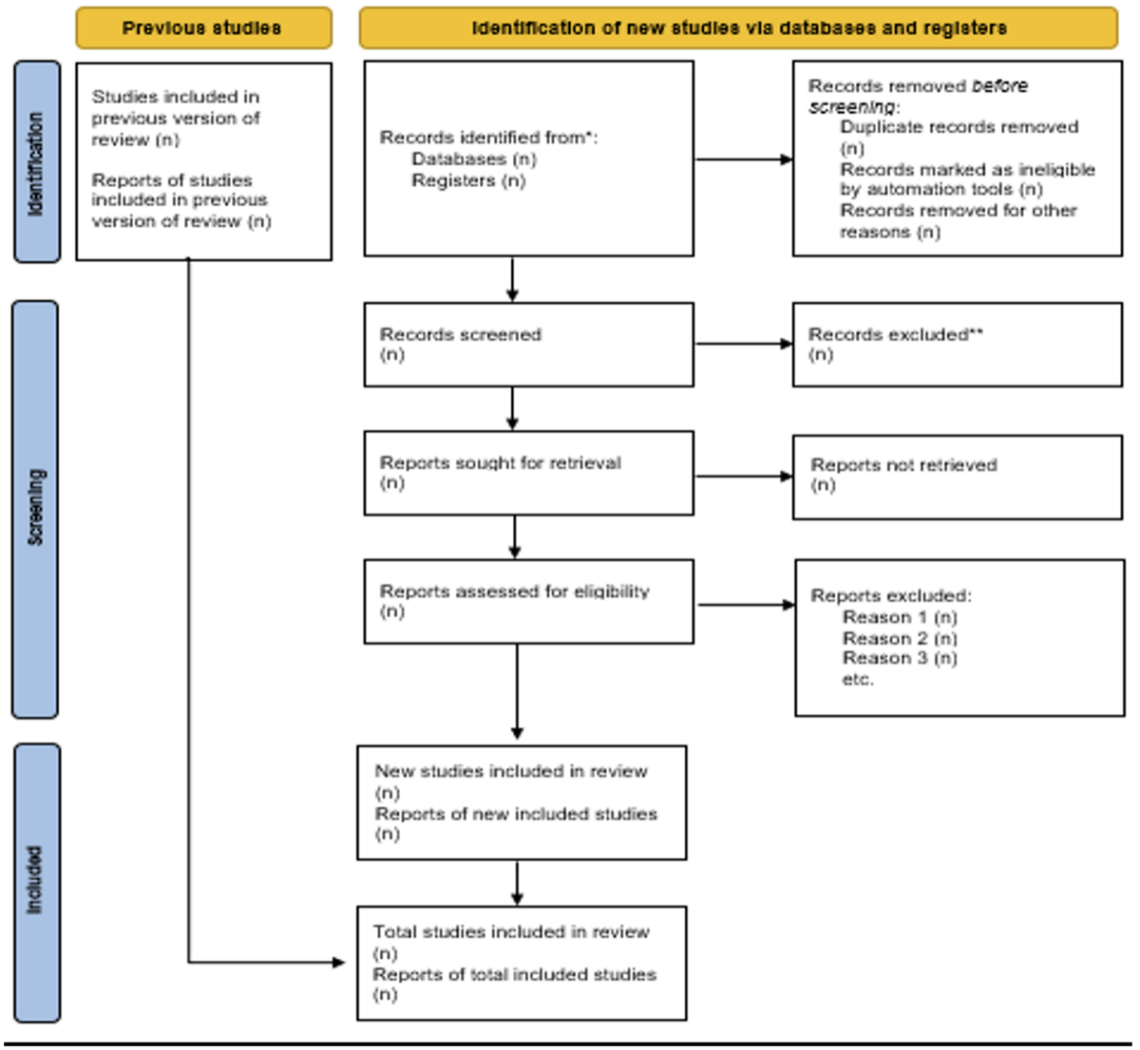
PRISMA (Preferred Reporting Items for Systematic Reviews and Meta-Analyses) flow diagram describing the study selection process (reproduced from Page et al [17]. This figure details the number of records identified, screened, assessed for eligibility, and included in the final analysis. It begins with the total number of articles retrieved through database searches (PubMed, Embase, and Cochrane Library), followed by the number of duplicates removed. Records are then screened by title and abstract, with exclusions noted. Full-text articles are reviewed for eligibility, with specific reasons for exclusion documented. The final number of studies included in the qualitative and quantitative synthesis is displayed at the bottom. This structured approach, based on PRISMA 2020 guidelines, ensures transparency and reproducibility in the literature selection process.

Each article identified through the search strategy described above will be reviewed by 4 independent researchers to ensure it meets the inclusion criteria. This process will be carried out step-by-step, including review of the title, abstract, and full text. A PRISMA (Preferred Reporting Items for Systematic Reviews and Meta-Analyses) flow diagram (Figure 1 [17]) will be included in the final review to further illustrate the screening process. Prior to initiating the formal screening process, all reviewers will undergo a training session and participate in a piloting exercise involving a random sample of articles. This calibration step is designed to ensure consistent interpretation of the inclusion and exclusion criteria and improve interreviewer reliability. Any discrepancies identified during piloting will be discussed and used to refine reviewer alignment before full screening begins. The screening and selection process will be managed using Rayyan, a web-based platform for systematic reviews. Rayyan will enable independent, blinded screening of titles and abstracts by at least two reviewers. Full-text screening will follow the same process. Disagreements will be resolved through consensus discussions, and if necessary, a third reviewer will adjudicate. This workflow will enhance transparency, minimize bias, and ensure a structured review process.

### Data Extraction and Main Data Elements

Four research team members will independently extract data from the articles accepted through the screening process to ensure a thorough and accurate review. Any discrepancies in the findings after data extraction will be discussed among authors and another independent team member to determine their relevance to the systematic review. The outcomes of the meta-analysis and systematic review will include the following: (1) occurrence of thromboembolic events during warfarin therapy, (2) occurrence of bleeding events during warfarin therapy, (3) all-cause mortality, (4) prosthetic endocarditis, (5) preoperative aortic valve replacement or repair, and 6) hemolytic anemia.

### Quality or Risk of Bias Assessment

All studies included in the final publication that meet the original inclusion criteria will be assessed using the National Heart, Lung, and Blood Institute (NHLBI) Quality Assessment Tool [18]. Randomized controlled trials will be assessed using the NHLBI Quality Assessment Tool for Controlled Intervention Studies, while noninterventional studies (eg, cohort and cross-sectional designs) will be evaluated using the NHLBI Quality Assessment Tool for Observational Cohort and Cross-Sectional Studies. The evaluation will be conducted according to the NHLBI criteria outlined in Textbox 2. The scoring protocol follow the model previously developed by Elks et al [19]. Two independent reviewers will evaluate each study, and discrepancies will be resolved through discussion or consultation with a third reviewer. To assess consistency between reviewers, the Cohen κ statistic will be calculated to quantify interrater agreement for each domain of the quality assessment. A κ value ≥0.75 will be considered excellent agreement, 0.40-0.74 moderate to good agreement, and <0.40 poor agreement. This additional layer of statistical validation will enhance the transparency and rigor of our bias assessment process.

**National Heart, Lung, and Blood Institute (NHLBI) Quality Assessment Tool used to evaluate risk of bias in included studies. This checklist includes multiple domains assessing internal validity, including participant selection, exposure and outcome measurement, confounding control, statistical analysis, and reporting clarity. Each item is rated as “yes,” “no,” “cannot determine,” or “not applicable” according to standardized NHLBI criteria. Two independent reviewers applied the tool to all included studies. Discrepancies were resolved by consensus or a third reviewer.**

**Quality Assessment of Controlled Intervention Studies: Criteria**

1. Was the study described as randomized, a randomized trial, a randomized clinical trial, or an RCT?
2. Was the method of randomization adequate (i.e., use of randomly generated assignment)?
3. Was the treatment allocation concealed (so that assignments could not be predicted)?
4. Were study participants and providers blinded to treatment group assignment?
5. Were the people assessing the outcomes blinded to the participants’ group assignments?
6. Were the groups similar at baseline on important characteristics that could affect outcomes (e.g., demographics, risk factors, co-morbid conditions)?
7. Was the overall drop-out rate from the study at endpoint 20% or lower of the number allocated to treatment?
8. Was the differential drop-out rate (between treatment groups) at endpoint 15 percentage points or lower?
9. Was there high adherence to the intervention protocols for each treatment group?
10. Were other interventions avoided or similar in the groups (e.g., similar background treatments)?
11. Were outcomes assessed using valid and reliable measures, implemented consistently across all study participants?
12. Did the authors report that the sample size was sufficiently large to be able to detect a difference in the main outcome between groups with at least 80% power?
13. Were outcomes reported or subgroups analyzed prespecified (i.e., identified before analyses were conducted)?
14. Were all randomized participants analyzed in the group to which they were originally assigned, i.e., did they use an intention-to-treat analysis?

### Strategy for Meta-Analysis

The statistical analysis for this meta-analysis will involve several key steps to assess the practical benefits of higher-intensity warfarin therapy for patients with MAVR and additional thromboembolic risk factors. First, data will be pooled using a random-effects model, as this approach accounts for potential variability between studies [20]. For continuous outcomes, such as INR level or left atrial dimension >50 mm, mean differences or standardized mean differences will be calculated. For dichotomous outcomes, such as bleeding or thromboembolic events, all-cause mortality, prosthetic endocarditis, preoperative aortic valve replacement or repair, and hemolytic anemia, we will compute risk ratios or odds ratios with 95% CIs. In addition to dichotomous outcomes, we will also extract time-to-event outcomes when reported, such as cumulative incidence rates at specific follow-up intervals or hazard ratios derived from survival analyses. If sufficient studies report comparable timeframes and event definitions, we will explore the feasibility of conducting a meta-analysis of time-to-event data using appropriate statistical models (eg, pooled hazard ratios). When heterogeneity in reporting precludes quantitative pooling, a narrative synthesis of time-to-event results will be included to provide additional context.

Subgroup analyses will explore potential differences based on factors such as additional thromboembolic risk factors (eg, atrial fibrillation, prior thromboembolism, hypercoagulable states, or severe left ventricular dysfunction), spontaneous echocardiographic contrast in the left atrium, significant vascular disease, and estrogen replacement therapy, as well as age, sex, and study design (randomized controlled trials vs observational studies). Sensitivity analyses will be conducted by excluding studies with a high risk of bias or small sample sizes to evaluate the robustness of the results. The heterogeneity of the results will be assessed using the *I*^2^ statistic, and if significant heterogeneity is detected, meta-regression and subgroup analyses will be performed to explore its sources. Publication bias will be assessed using funnel plots and the Egger test [21]. Statistical significance will be defined as a 2-sided *P* value of less than .05. The Comprehensive Meta-Analysis Package (version 4.0; Biostat Inc) will be used for data analysis.

### Data Analysis and Presentation

Assuming sufficient data are extracted, the data will be presented in a detailed table, accompanied by a final summary discussing the analysis performed. A data extraction form will be created and reviewed by all investigators before analysis. The articles included in the publication will be listed as horizontal rows in the table, and the variables agreed upon by the team will serve as columns. Quantitative analysis will be conducted on all variables extracted from the available data.

## Results

A PRESS review of the search strategy with an academic librarian was conducted in December 2024. The development of the search strategy began in December, and the final strategy was completed by the team of investigators in January 2025. Database queries and title screenings will begin in February 2025, with abstract screenings scheduled for February and March 2025. Full-text retrieval will occur from March to May 2025. Data extraction, synthesis, and risk of bias assessment will be carried out in May and June 2025. Data analysis and manuscript drafting will take place during July and August 2025. The findings will be summarized, organized, and submitted to a peer-reviewed journal by September 2025. The primary goals of this effort are to assess the utility of higher INR goals in patients with MAVR with additional thromboembolic risk factors, balancing the benefits and risks of harm, and to inform the development of future guidelines for medical practice. A timeline is presented in Table 2.

**Table 2:** Project milestones and timelines.

Tasks	December 2024	January 2025	February 2025	March 2025	April 2025	May 2025	June 2025	July 2025	August 2025	September 2025
1. Protocol development	✓									
2. Design of search strategy	✓	✓								
3. Title screening			✓							
4. Abstract screening			✓	✓						
5. Full-text screening				✓	✓	✓				
6. Data extraction						✓	✓			
7. Synthesis and risk of bias assessment						✓	✓			
8. Data analysis								✓	✓	
9. Manuscript drafting								✓	✓	
10. Submission and peer review										✓

## Discussion

### Overview

Our systematic review will be a comprehensive analysis of the relevant literature to synthesize the need for higher-intensity anticoagulation therapy in patients with MAVR and additional thromboembolic risk factors. We anticipate that the results of our analysis will align with current guidelines, recommending higher INR goals (3.0-3.5) for this group compared to the standard INR goals (2.5-3.0) for patients without additional risk factors. We will analyze each thromboembolic risk factor, such as atrial fibrillation, history of thromboembolism, hypercoagulable states, and left ventricular systolic dysfunction, to evaluate their individual contributions to thromboembolic events in this population. In addition to the thromboembolic risk factors highlighted in the 2020 ACC/AHA and 2012 ACCP guidelines, we will also explore other potential risk factors, including left atrial dimension >50 mm, spontaneous echocardiographic contrast in the left atrium, significant vascular disease, and concurrent estrogen replacement therapy. In addition to major and minor thromboembolic and bleeding events as primary end points, we will examine all-cause mortality, prosthetic endocarditis, preoperative aortic valve replacement or repair, and hemolytic anemia as secondary end points to assess both the positive and negative outcomes of higher-intensity anticoagulation therapy in this group.

### Significance

Although studies have examined the optimal INR goals for patients with MAVR, no previous systematic review or meta-analysis has specifically addressed the group with additional thromboembolic risks. As our study will be the first to focus on thromboembolic risk factors in patients with MAVR related to anticoagulation therapy, it may help validate the current guidelines, establish new ones, and guide future research in this patient population. In addition to primary RCTs, our systematic review will include secondary or follow-up RCTs of the original studies. This broader inclusion criterion will enhance subgroup analyses and provide a more comprehensive understanding of the safety profile of anticoagulation therapy in patients with additional thromboembolic risk factors. This study will balance the benefits of thromboembolic prevention against the harms of bleeding associated with higher-intensity warfarin therapy in this group. Since the majority of MAVR candidates already have significant cardiac conditions, such as atrial fibrillation or left ventricular systolic dysfunction due to chronic severe aortic valvular disease, most patients with MAVR are naturally at high thromboembolic risk. Given the lack of supporting data, many physicians and institutions continue to follow standard anticoagulation therapy guidelines despite the presence of additional thromboembolic risk factors. We anticipate that our study will benefit the majority of patients with MAVR, as most are at high thromboembolic risk for the reasons outlined above.

### Limitations

Our study will have several limitations. First, the selected articles were conducted in different settings, with varying demographics, primary and secondary end points, time periods, follow-up periods for INR checks, and INR goals. This variability in methodologies will introduce inherent heterogeneity. Additionally, there is a potential for reviewer bias during article selection. To mitigate this, we will implement the following strategies: (1) an individualized filtering process with 4 independent reviewers, with the first author overseeing the entire screening process to resolve any disagreements; (2) full transparency of methods, with subject matter experts providing input at each step of the systematic review; and (3) a standardized risk of bias assessment for every article. There is also a risk of publication bias in the article selection process, as studies with small sample sizes or insignificant results are less likely to be published, potentially causing relevant work to be overlooked. An additional limitation of our review is the restriction to English-language articles. This decision was made due to resource constraints and to ensure accuracy in data extraction and analysis, as our review team does not have access to certified medical translators. While this may introduce language bias and potentially exclude relevant studies published in other languages, previous meta-epidemiological studies have shown that excluding non-English articles often has a limited effect on the overall conclusions in many clinical domains [22]. Nonetheless, this language restriction may impact the comprehensiveness and global applicability of our findings, and we acknowledge it as a potential source of bias. Finally, given the variable study methodologies, we may encounter diverse outcomes of interest. While we hope our study will provide valuable insights and future directions on this topic, the possibility remains that the results may not reach statistical significance.

### Dissemination Plan

Our systematic review will be submitted as a manuscript to a peer-reviewed journal by September 2025. Abstracts based on this study will be created, submitted, and presented at academic conferences.

### Relevance to Practice and Policy

This systematic review protocol was developed by a multidisciplinary team of clinician-researchers specializing in cardiovascular medicine, internal medicine, and health outcomes research. Their clinical experience managing patients with MAVR directly informed the formulation of the research question and outcome selection, ensuring alignment with frontline clinical challenges.

To further enhance the applicability of the findings, we plan to engage a broader group of end users during the dissemination phase. This includes clinicians, patient advocacy groups, and policymakers. Presentations at cardiology and health services conferences, along with institutional and policy-focused briefings, will serve as platforms to gather feedback and support translation of findings into practice. We also aim to develop accessible summaries of the results tailored to patients and decision-makers to maximize the impact of this review on future guideline development and individualized care strategies.

### Conclusions

This systematic review will provide a comprehensive evaluation of the safety of high-intensity anticoagulation therapy in patients with MAVR and additional thromboembolic risk factors, including atrial fibrillation, previous thromboembolism, a hypercoagulable state, or severe left ventricular dysfunction, as outlined in the 2020 ACC/AHA guidelines. We will also broaden the scope to include other thromboembolic risk factors, such as left atrial dimension >50 mm, spontaneous echocardiographic contrast in the left atrium, significant vascular disease, and estrogen replacement therapy. The primary end points will include major or minor thromboembolic and bleeding events, while secondary end points will include all-cause mortality, prosthetic endocarditis, preoperative aortic valve replacement or repair, and hemolytic anemia. Our study will also generate a qualitative synthesis of evidence regarding the risks of thromboembolism and bleeding across different INR goals in this patient population. Ultimately, our findings may guide future research and either support or modify current guidelines.

## Abbreviations

ACC: American College of Cardiology
ACCP: American College of Chest Physicians
AHA: American Heart Association
AVR: aortic valve replacement
INR: international normalized ratio
MAVR: mechanical aortic valve replacement
NHLBI: National Heart, Lung, and Blood Institute
PECOS: population, exposure, comparison, outcome, and study design
PRESS: Peer-Reviewed Electronic Search Strategy
PRISMA: Preferred Reporting Items for Systematic Reviews and Meta-Analyses
RCT: randomized controlled trial
